# Experimental Models, Induction Protocols, and Measured Parameters in Dry Eye Disease: Focusing on Practical Implications for Experimental Research

**DOI:** 10.3390/ijms222212102

**Published:** 2021-11-09

**Authors:** Md. Mahbubur Rahman, Dong Hyun Kim, Chul-Kyu Park, Yong Ho Kim

**Affiliations:** 1 Gachon Pain Center and Department of Physiology, Gachon University College of Medicine, Incheon 21999, Korea; mahbubpharmvet@gmail.com (M.M.R.); pck0708@gachon.ac.kr (C.-K.P.); 2 Gil Medical Center, Department of Ophthalmology, Gachon University College of Medicine, Incheon 21565, Korea; amidfree@gmail.com

**Keywords:** dry eye, pathophysiology, in vitro models, in vivo models, therapeutic protocol, measured parameters

## Abstract

Dry eye disease (DED) is one of the major ophthalmological healthcare challenges worldwide. DED is a multifactorial disease characterized by a loss of homeostasis of the tear film, and its main pathogenesis is chronic ocular surface inflammation related with various cellular and molecular signaling cascades. The animal model is a reliable and effective tool for understanding the various pathological mechanisms and molecular cascades in DED. Considerable experimental research has focused on developing new strategies for the prevention and treatment of DED. Several experimental models of DED have been developed, and different animal species such as rats, mice, rabbits, dogs, and primates have been used for these models. Although the basic mechanisms of DED in animals are nearly identical to those in humans, proper knowledge about the induction of animal models is necessary to obtain better and more reliable results. Various experimental models (in vitro and in vivo DED models) were briefly discussed in this review, along with pathologic features, analytical approaches, and common measurements, which will help investigators to use the appropriate cell lines, animal, methods, and evaluation parameters depending on their study design.

## 1. Introduction

Dry eye disease (DED) is a multifactorial disease characterized by inefficient tear secretion or increased evaporation of tear film, which hamper tear osmolarity and ocular surface lubrication, increase shear forces under the eyelids, and finally trigger inflammation and pathologic complexity. This can cause an imbalance of electrolytes, mucins, and proteins and damage the conjunctival and corneal epithelial cells and nerve fibers. It is also known as dry eye syndrome, keratoconjunctivitis sicca, and chronic inflammatory ocular surface disease. The primary clinical signs of DED are eye irritation, increased tear osmolarity, impairment of ocular surface epithelia, tear film instability, and blurred and fluctuating vision. Chronic inflammation, irritation, and pain induced by DED have a marked negative influence on the quality of life, hampering visual performance and the ability to perform daily tasks (e.g., reading and driving). These detrimental impacts on functioning lead to anxiety and depression [1]. Aged populations (approximately 10–20%) are more prevalent in DED [2]. It was surveyed that 1.68 million men over the age of 50 suffer DED in the United States, and this number is likely to grow to 2.79 million by 2030 as life expectancy elevates [3]. However, females of all age groups have a greater tendency to have DED than males, with DED prevalence increasing with age according to Drew et al. [4]. It was also reported that over 3.23 million women suffer from DED [5]. Therefore, considering several survey reports [5,6,7], DED is a significant health concern and has global socio-economic impacts.

The animal model in experimental biomedical research refers to a simplified representation of disease condition, hoping that obtaining information can be transferred to actual human or animal diseases. Nowadays, experimental basic animal research for application of medicinal practice and drug discovery has gained reliability. It became popular after its introduction in the National Institutes of Health Roadmap initiative [8,9]. Experimental animal research has also been widely applied in ocular medicine. The DED model is one of them. Most reports, including reviews [1,4,10,11,12,13], have been focused on the discovery of drugs that would be relevant to study the human DED pathogenesis and evaluate the therapeutic outcomes of future pharmacological treatments. However, induction methods are important when evaluating the function of some mechanisms discovered in animal models in the pathophysiology of human disorders and when applying to discover new treatments for these conditions. Therefore, this review study focused on the design of experimental models—especially on the techniques, types of induction methods, types of cell lines for in vitro models, types of animals for in vivo models, dosages of drugs, frequency of drugs, pathological mechanisms—and cellular cascades of DED, and animal modeling. Evaluation methods and parameters in experimental DED such as specific for clinical signs tear deficiency, ocular surface structural damage, and molecular alterations such as inflammatory cytokines, apoptosis, fibrosis, or angiogenesis-related markers were also described briefly. In addition, this review also briefly described the anatomical location physiology function and pathological condition of DED related glands tissue or cells (lacrimal glands, meibomian glands (MBGs), conjunctival and corneal epithelium, goblet cells, tear film, Harderian gland, and nictitating membrane (NM)) for a better understanding of the pathophysiological mechanisms of experimental DED. We hope this study will provide clear information on experimental DED.

## 2. Pathophysiology of Dry Eye Disease (DED)

First, understanding the ocular pathophysiology is crucial for understanding the mechanisms of induction methods. The ocular surface is an exposed part of the eye. Therefore, the fluidity of this part evaporates owing to multiple environmental factors, including variable airflow, temperature, and humidity. Maintaining continuous lubrication in this part is challenging for maintaining normal health and performing normal functions. Lubrication depends on efficient tear production and turnover. The main and accessory lacrimal glands, MBGs, tear film, goblet cells, all ocular surface secretory cells, smooth pathways of lacrimal outflow, and conjunctival and corneal epithelia work simultaneously as a lacrimal functional unit (LFU) to maintain the tear film and protect the transparency of the cornea and the integrity of the ocular surface [12]. LFU is affected by many factors, such as nerve connections and hormones. Disturbances to any part, such as the afferent sensory nerves, efferent autonomic and motor nerves, tear-secreting glands, and lipid meibum-secreting glands, may lead to DED [1].

### 2.1. Lacrimal Glands

**Figure 1:**
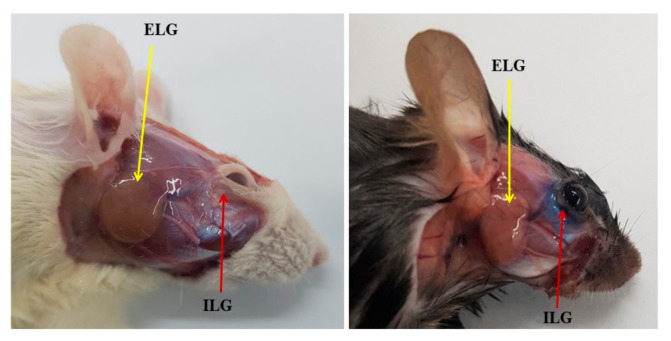
Location of the external (ELG) and internal lacrimal glands (ILG) in Sprague–Dawley rat (**left**) and C57BL/6 mouse (**right**).

The lacrimal glands are exocrine glands. There are two types of glands: main and accessory lacrimal glands. The human main lacrimal gland is paired with each eye; it is situated in the upper lateral region of each orbit, in the lacrimal fossa of the orbit formed by the frontal bone. In rodents, including rats, mice have two pairs of lacrimal glands: the smaller intraorbital (infraorbital) gland is located slightly below the eye socket, and the larger exorbital lacrimal gland is located outside the eye socket, ventrally in front of the acoustic duct [14] (Figure 1). Lacrimal glands secrete aqueous tears composed of water, electrolytes, mucus, and proteins (lysozyme, immunoglobulins, lacritin, and cytokines) and form the tear film’s aqueous layer [13]. There are two types of accessory lacrimal glands: Wolfring’s/Ciaccio’s glands and Krause’s glands, as shown by Conrady et al. [15]. The glands of Wolfring and Ciaccio are small tubular-sized glands in the lacrimal caruncle of the eyelid and upper border of the tarsus, approximately in the middle between the extremities of the tarsal glands [16,17]. Approximately two to five glands are present in the upper eyelid. In contrast, Krause’s glands are comparatively smaller in shape and more numerous than Wolfring’s glands and are situated along the superior and inferior fornices of the conjunctival sac [18]. There are approximately 40 Krause glands in the upper eyelid and around six to eight in the lower lid [19]. Their function is to produce tears secreted onto the surface of the conjunctiva. However, the lacrimal gland epithelia also secrete small soluble mucin MUC7 [20].

### 2.2. Meibomian Glands (MBGs)

These are a special type of sebaceous gland, a holocrine-type exocrine gland arranged vertically within the tarsal plates of the upper and lower eyelids. MBGs are also known as the tarsal glands. Approximately, the upper eye lid tarsus contains about 30–40 glands, and the lower eye lid tarsus contains 20–40 MBG glands [21,22]. MBG primarily secretes lipid meibum, which is an oily substance that forms the lipid layer of the tear film and plays a pivotal physiological role in maintaining tear film stability, preventing evaporation of the ocular surface, preventing tear film contamination, and reducing surface tension [23,24]. In addition, lubricating molecules such as mucinous glycoprotein and lubricin synthesized by the MBG have been reported [24]. These molecules play an important role in the tribology of the eye and decrease the friction between the eyelid and the ocular surface [24]. The disturbances of MBG trigger abnormalities in the lipid layer of the tear film, leading to evaporative dry eye. This situation is defined as MBG dysfunction (MGD), which is one of the major causes of DED [22,23,25].

### 2.3. Conjunctival and Corneal Epithelium, Goblet Cells, and Mucins

The conjunctiva occupies two-thirds of the ocular surface, from the corneal rim to the lid margin. Both corneal and conjunctival epithelial cells participate in producing different mucins on the ocular surface, but the conjunctival goblet cells are the main mucin-producing cells [26]. Goblet cells are specialized epithelial cells that are present in the mucosal tissues along the body. The major function of these cells is to produce and secrete mucins that hydrate and lubricate mucosal surfaces [20]. In addition, the highly stratified corneal epithelium also produces mucins [13]. Mucins are highly glycosylated glycoproteins composed of a protein core and multiple side chains [27]. Mucins contain two types of transmembrane and secretory proteins. According to the polymer-forming ability, secretory mucin is further subdivided into gel-forming and soluble mucins. Mucins that have been identified in the eye are MUC1, MUC2, MUC4, MUC5AC, MUC7, MUC13, MUC15, MUC16, and MUC17 [2,28]. One of the most common large gel-forming secretory mucins in the conjunctiva is MUC5AC [26]. Mucin maintains tears’ rheological properties and viscosity, providing a lubricative function during rapid and high share movements of the lid on the globe and vice versa, thereby protecting the eye from frictional damage. Therefore, a lack of mucin or goblet cells reduces tear breakup time. It also helps spread oil from MBG secretion, thus maintaining the lipid layer of tear film integrity, hence checking evaporation [29].

### 2.4. Tear Film

The tear film (TF) is a special form of the extracellular matrix part of the ocular surface approximately 3–8 μm thick [2,30] and 3 μL in volume in humans [30]. The thickness of TF may vary depending on the animal and the measuring methods (Table 1). It is essential for lubrication between the inner surface of the eyelids and cornea to rationalize the frictional forces during blinking, provide an optically smooth medium, and protect the ocular surface from potentially pathogenic microbes [24,30]. It comprises three layers: the outermost lipid layer, the middle aqueous layer, and the innermost mucous layer [13,15]. The middle aqueous layer is a major part of the TF [31].

The lipid layer of TF is produced by the MBG secretion. It is a bilayer and is the thinnest part of TF, approximately 50–100 nm in humans [30]. The upper part of the bilayer served as the air–TF interface, which is comprised of non-polar lipids (e.g., wax esters, cholesterol, and steroid esters) and lesser amounts of polar lipids such as O-acyl-ω-hydroxy fatty acids and phospholipids, which interact adjacent to the aqueous layer [24,30]. The vital role of this layer is to retard the evaporation of the underlying aqueous layer in the open eye, but it also maintains surface tension, elasticity, viscosity, and systematic pecking order, which aids the tear film in preserving the ocular surface integrity [13,30]. The middle aqueous layer of TF is produced by the main and accessory lacrimal gland secretions. Aqueous fluids contain water, electrolytes, and various proteins, including immunoglobulins, cytokines, and growth factors [13,30]. The conjunctival epithelium also contributes to electrolytes and water in tears [30]. The aqueous phase supplies oxygen and nutrients to the underlying adjacent avascular conjunctival and corneal tissue and rinses toxins, epithelial debris, and foreign bodies. It plays an important role in cell signaling and rehabilitation of the ocular surface during disease conditions. The aqueous phase contains electrolytes that maintain tear osmolarity [13,30]. The innermost mucous layer of TF is mainly formed by mucins, immunoglobulins, salts, urea, enzymes, glucose, and leukocytes [13]. Mucins are produced by the goblet cells in the conjunctival and corneal epithelium, and some transmembrane mucins are projected into the aqueous phase of TF from corneal and conjunctival epithelial cells [13,30], thereby stabilizing the TF. Soluble mucins connect with the transmembrane mucins and form a stable glycocalyx layer over the epithelium. The stratified corneal epithelium also produces O-glycosylated transmembrane mucins, contributing to the glycocalyx region. The microvilli of the superficial epithelium of the cornea also participate in the anchorage of the tear film [13].

### 2.5. Harderian Gland and Nictitating Membrane (NM)

The Harderian gland also plays an important role in the ocular surface homeostasis in some animals besides the lacrimal and MBG. Rodents (rats, mice, hamsters, gerbils, and guinea pigs) have the most developed Harderian gland compared to other animals. It is situated around the posterior half of the eyeball and occupies a significant part of the orbit in rodents [36]. The secretion component varies according to the different animal species; for example, mucolipidic in amphibians, mucoserous in reptiles, serous in snakes, and lipidic in bird secretions. In mammals, including rats, their secretions contain three components: lipids, indolic products, and porphyrins [37]. Its secretion also plays a role in lubricating the eye, especially by easing the NM in mammals and amphibians [36,38].

The NM, also known as the third eyelid, is present in most animals around the medial canthus, while humans and anthropoids maintain a vestigial remnant of this organ called plica semilunaris [39,40]. The physiological function of the plica semilunaris in humans is less in human, but the NM in animals contributes to a healthy animal eye by producing and distributing tears, secreting immune proteins, removing ocular debris, and acting as a mechanical barrier [40,41]. Therefore, removing the NM and the Harderian gland has been used to induce dry eye models [42,43,44,45].

## 3. Classification of Dry Eye

**Figure 2:**
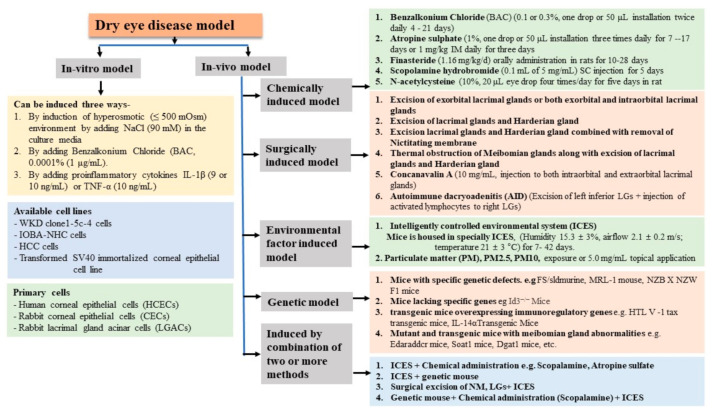
Classification of various dry eye models.

DED is mainly classified into two types: (a) aqueous tear-deficient DED and (b) evaporative DED. Aqueous tear-deficient DED is further classified into two types: Sjogren syndrome and non-Sjogren syndrome. Sjogren syndrome is mainly associated with autoimmune diseases such as rheumatoid arthritis, systemic lupus erythematosus, Wegener’s granulomatosis, and other diseases (such as systemic sclerosis and primary biliary cirrhosis). Non-Sjögren syndrome is associated with lacrimal diseases (such as congenital alacrima, acquired primary lacrimal gland disease, and sarcoidosis), lacrimal gland obstruction such as trachoma, cicatrical pemphigoid erythema, and multiforme burns. On the other hand, evaporative DED may be caused by oil deficiency (absent glands MGD, distichiasis, blepharitis, obstructive MBGs), lid-related diseases (blink abnormalities, aperture abnormalities, lid surface incongruity), allergic conjunctivitis, or xerophthalmia (vitamin A deficiency) [46]. However, in an experimental study, the DED model can be broadly induced in two ways: in vitro and in vivo DED models. Furthermore, in vivo disease models can be classified into five ways: chemically induced model, surgically induced model, environmental factor induced model, genetic model, and combined methods induced model (Figure 2).

### 3.1. In Vitro Dry Eye Model

In preclinical studies, an in vitro experiment provided tight control of the microphysio-pathological environment of the animal body, which has the most obvious benefit of reducing the requirement of animal testing and is ethically more appropriate. It is also beneficial for obtaining quick, efficient, reliable, and robust results in the early stages of candidate drug screening. In DED, some established in vitro models have also been established: the Wong Kilbourne derivative of Chang conjunctival epithelial cell line (WKD; clone 1–5c-4) [47,48,49,50,51]; immortalized normal human conjunctival cell line (IOBA-NHC) [47,52,53]; human corneal epithelial cells (HCECs), a human transformed SV40 immortalized corneal epithelial cell line [54,55], and human conjunctival cell line (HCC) [56]. In vitro models with primary cell culture are also available: human corneal epithelial cells (HCECs) [57], rabbit corneal epithelial cells (CECs), or rabbit LG acinar cells (LGACs) [58]. The induction of DED-like symptoms in in vitro culture can be performed by the induction of hyperosmotic (500 mOsM) media in the culture media by the addition of 90 mM sodium chloride in the culture medium, which triggers inflammatory-like symptoms [47,48,55,57,59]. As in the in vivo experiment, benzalkonium chloride (BAC) (0.0001% (1 μg/mL) was also used to induce inflammatory cascades [47,48,49,50,51,52]. In addition, recombinant pro-inflammatory cytokine interleukin (IL)-1β (9 or 10 ng/mL) or tumor necrosis factor alpha (TNF α) (10 ng/mL) can be used to induce inflammatory cascades in the culture medium for 24 h or 48 h [53,58]. Cell culture methods and experimental schedules are described briefly in Table 2.

### 3.2. In Vivo Dry Eye Models

In preclinical studies, in vivo testing is a major part of safety assessment and is a regulatory requirement prior to clinical trials as it provides a similar biological environment and properties to humans. Therefore, it is more realistic and reliable than in vitro studies and provides important data for function validation, clinical trials, and approval by the FDA and regulatory authorities. In vivo experimental DED is generally induced in laboratory animals by several methods, including chemical, surgical, controlled environmental system, genetic (immunological) manipulations, and combined methods. Most of the experiments in DED are carried out in rabbits and rodents, although some studies are still performed on other animals, such as monkeys and pigs. Among these models, the chemically induced model is the most commonly used technique.

#### 3.2.1. Chemically Induced Dry Eye Model

##### Benzalkonium Chloride (BAC)

BAC is an organic salt, a quaternary ammonium compound, and is a type of cationic surfactant. It is also known as the alkyl dimethyl benzyl ammonium chloride. It is mainly used in three ways: an antimicrobial, a cationic surfactant, and a phase transfer agent [60]. In ocular medicine, it is widely used as a preservative in eye drops, with typical safe concentrations of 0.004–0.01%, which are most commonly used at a concentration of 0.01% in ophthalmic preparations [61]. However, experimental and clinical studies have found that the long-term use of topical drugs with BAC may alter ocular surface integrity, tear film instability, inflammation, and loss of goblet cells, conjunctival squamous metaplasia, epithelial apoptosis, subconjunctival fibrosis, and the potential risk of failure for further glaucoma surgery [62]. Liang et al. [63] showed that short and repeated exposure to 0.02% BAC induced a large number of inflammatory cells in the rabbit corneal epithelial basal layer and stroma [64]. They experimentally applied 0.01%, 0.05%, or 0.1% twice daily to one eye each of rabbits for 4 days and showed that BAC at 0.1% resulted in significant increases in central corneal thickness, endothelial CF permeability, endothelial cell damage with dislocation of ZO-1, and disruption of peri-junctional actomyosin ring (PAMR) compared with those in control eyes with other doses. In addition, Pauly et al. [65] (2005) applied 0.01%, 0.1%, 0.25%, and 0.5% to rat corneas for 11 days and found that 0.25% and 0.5% induced increased corneal thickness, loss of goblet cells, reversible corneal inflammation, and persistent neovascularization. Since then, experimental evidence has shown that the toxic effect of BAC on the ocular surface is mainly related to its concentration. Nonetheless, 0.1% BAC is commonly used to induce the DE model. Li et al. [66] applied 0.1% BAC twice daily for 2 weeks, 3 weeks, 4 weeks, or 5 weeks and concluded stable, sustainable DE induced by a 5-week BAC treatment group. Furthermore, the effects of different concentrations of BAC (0.01%, 0.05%, 0.1%, 0.2%, 0.3%, 0.5%, and 1.0%), different frequencies of topical application (two to four times daily), and different lengths of treatment (1–4 weeks) were evaluated for their effects on the rabbit ocular surface using the Schirmer test, ocular surface staining, CIC, and microscopy in the study by Xiong et al. [67]. It was found that twice-daily topical administration of 0.1% BAC drops for 2 weeks was the optimal procedure to induce dry eye syndrome in white New Zealand rabbits. However, using lower BAC concentrations was insufficient to induce tear deficiency over 2 weeks, while higher concentrations and multiple applications induced serious ocular surface damage and corneal ulcer, vascularization, and scarring, similar to chemical burns. Nonetheless, using 0.2% BAC for 2 weeks was also found to induce dry eye in mice [68].

Finasteride, atropine sulfate, scopalamine hydrobromide, and N-acetyl cysteine were also used to induce DED. The doses and experimental protocol are shown in Table 3. Androgen deficiency is one of the main causes of dry eye, as androgen plays a vital role in the regulation of lacrimal gland secretory functions [69], consequently inducing decreased tear secretion, ocular surface damage, and lacrimal gland inflammation, which are the characteristics of dry eye [70]. Finasteride is a 5α-reductase inhibitor that works by decreasing the production of dihydrotestosterone; therefore, it is called antiandrogen. In animal studies, it was also shown that finasteride administration decreased testosterone levels and DED [71].

Atropine is an anticholinergic antimuscarinic drug that inhibits the parasympathetic nervous system. Therefore, the topical administration of atropine sulfate has been used to induce dry eye models because of its ability to decrease tear production [72,73,74]. Moreover, as a muscarinic receptor blocker [75] and parasympathetic nerve inhibitor [76], scopolamine hydrobromide is also used to induce dry eye models. Scopolamine reduces the action of the central nervous system, such as atropine, by inducing nerve paralysis, mydriasis, and inhibition of secretion [75]. N-acetylcysteine (NAC) is a derivative of the amino acid L-cysteine that acts as a mucolytic agent that breaks mucoprotein disulfide bonds, thereby converting low molecular weight mucin molecules. Multiple or long-time topical exposure 0.1 M or 10% NAC induces the loss of the conjunctival mucus layer or mucin-like substances in rabbit eyes [77,78]. In addition, corrosive 50% trichloroacetic acid is also used to induce the dry eye model [45].

#### 3.2.2. Surgically Induced Dry Eye Model

Another way to induce experimental DE is via excision of the ocular secretory glands. The lacrimal glands, MBGs, Harderian gland, and NM work together to maintain the tear film’s moisture and lubrication of the ocular surface. Therefore, these parts are the surgical targets for inducing the dry eye model. After removing the lacrimal gland, dry eye occurred due to fluid deficiency [14], and the Harderian gland was removed together with severe fluid deficiency. Blocking the MBG induces oil deficiency in the lacrimal fluid of the ocular surface; consequently, the fluid evaporates easily and induces dry eye. Surgically, it can be induced by removing the lacrimal gland [14,95]; removal of the lacrimal gland (LG), Harderian gland (HG), and NM [42,43,44,45] or by thermal obstruction of MBG along with removal of the lacrimal and Harderian glands [96].

DED can also be induced by another surgical technique by excision of the left inferior LGs for the isolation of purified epithelial cells and peripheral blood from each rabbit to collect peripheral blood lymphocytes. Activated lymphocytes are injected into the right LGs to induce autoimmune dacryoadenitis (AID), which results in tear deficiency. Consequently, DED is induced within 2 weeks of disease [97,98].

Concanavalin A (Con A) jack bean plant-derived lectins when injected into the body bind to various glycoproteins and activated lymphocytes, trigger inflammatory cascades, and cause tissue injury [99,100]. Therefore, Con A injection into the lacrimal gland allowed the study of the pathophysiology of immunologically mediated lacrimal gland-related DED [100,101,102,103,104]. Similarly, injection of botulin toxin B into the lacrimal gland also induced DED [105]. The experimental methods are listed in Table 4.

#### 3.2.3. Environmental Factors-Induced DED Model

Another way to induce experimental DE is by applying an ICES [109,110]. An ICES is an artificial environment in which temperature, humidity, and airflow are regulated and recorded. This environment causes a deficiency of ocular surface fluidity by quick evaporation, in which dry eye can be induced due to critical environmental factors. However, the environmental factors and time of induction vary from article to article and are displayed in Table 5, which would be very helpful in ICES-induced experimental evaporative DED. Furthermore, the prevalence of DED in fine dust particulate matter-exposed persons is very high [111,112]. Recently, researchers found that the exposure of fine dust particulate matter induced DED in rodents [112,113,114].

#### 3.2.4. Genetically Engineered Dry Eye Model

Genetic models, genetic mutations, or modifications are performed in animals to develop spontaneous disease symptoms. Most genetic models represent Sjogren’s syndrome (SS), which is a chronic autoimmune disorder characterized by disease conditions, including keratoconjunctivitis sicca (dry eyes), xerostomia (dry mouth), and rheumatic arthritis along with vascular, dermatologic, hepatobiliary, renal, gastrointestinal, and nervous system dysfunctions [118]. Although SS genetic mice display a partial DED model, these models provide important information for understanding the molecular mechanisms of apoptosis, dysregulation of lacrimal gland homeostasis, and hyperosmolarity-related ocular surface dysregulation. The available SS mouse models include non-obese diabetic (NOD) mice, IL-2Rα (CD25) knockout mice, NZB/NZW-F1 mice, MRL-1 pr/1 pr mouse, TGF-β1 knockout mouse, thrombospondin-1 (TSP-1)-deficient mice, homozygous for the alymphoplasia (aly) mutation mouse, NFS/sld mouse, IQI/Jic mouse, and Id3-deficiency mouse [119,120,121]. Among these murine models, NOD mice (C57BL/6.NOD-Aec1Aec2 mice, NOD.H2b mice, NOD.Aire Knockout mice, and NOD.B10.H2b mice) are widely used because of their clinical relevance to autoimmune diseases [120,121,122]. In an experimental study, the identification of DED is important for the selection of therapeutic schedules, as listed in Table 6.

#### 3.2.5. Combining Methods

To increase the induction time of dry eye, two or three methods are combined to aggravate ocular surface dysfunction. For example, when C57BL/6 mice were used to induce DED, 21 days [115,116,117] and 42 days [110] were required (Table 5). However, when scopolamine was used along with ICES, DED was confirmed on day 9–14 [75,128,129,130,131,132]. Likewise, the topical application of atropine and ICES was used, and DED was induced after 48 h [130]. Surgically dissected NM rabbit were housed in low humidity and higher air flow-controlled environment to induce DED [133]. Interestingly, three factors, i.e., genetic mouse, chemical administration, and ICES, were also applied [122,134,135] (Table 7).

## 4. Evaluation of DED Severity and Therapeutic Efficacy of Candidate Drugs in an Experimental Model

Several parameters are used in experimental studies to evaluate the severity of DED and therapeutic efficacy of candidate drugs. However, not all of these are used in one experiment, which are based on the ocular abnormalities that occur following DED and therapeutic targets of the candidate drugs. Some tests are specific for clinical signs, some are specific for tear deficiency, and some are specific for ocular surface structural damage, along with molecular alterations such as inflammatory cytokines, apoptosis, fibrosis, or angiogenesis-related markers (Table 7).

### 4.1. Inflammatory Index and Clinical Scoring

Both inflammatory index and clinical scoring can be used in rabbits or large animals, but only the inflammatory index is used in rodents. The inflammatory index can be assessed as described previously [83,136] by considering the following criteria: ciliary hyperemia (0–3), central corneal edema (0–3), and peripheral corneal edema (0–3). The total inflammatory index results were measured for each criterion from each individual animal and divided by a factor of 9 (Table 8).

In disease models, clinical signs are important criteria for accepting experimental research as a viable means of declaring scientific validity and practical reliability similar to an actual disease. Moreover, the experimental clinical criteria should be met by measurement scores for easy differentiation among the experimental groups. However, there is no gold standard for clinical scores in the dry eye model. The severity score was modified from that of Eaton et al. [137] and Silva et al. [74]. The score varies from 0 to 16 and is defined as the sum of the individual scores graded from conjunctival hyperemia (0–3), conjunctival swelling (chemosis) (0–4), conjunctival discharge (0–3), corneal opacity (area) (0–4), and corneal vascularization (0–2). Ophthalmologic examinations can be performed using a portable slit lamp and visual examination. Clinical signs should be scored before induction of DE, prior to treatment, and then several times at time intervals until the end of the experiment to evaluate the therapeutic efficacy of drugs (Table 8).

### 4.2. Tear Deficiency-Related Tests

#### 4.2.1. Schirmer’s Test and Phenol Red Thread (PRT) Test

Schimer’s test and the PRT test are similar types of experiments that are used to evaluate tear secretion in response to both basal non-reflex secretion and conjunctival stimulation in DED in conscious animals without anesthesia. Schimer’s test was first introduced by German ophthalmologist Otto Schirmer over 100 years ago, and the PRT test was conducted by Japanese scientists in 1982 [151]. Comparatively, the PRT test is more sensitive and quicker than the Scimer strips. Only 15–60 s is required to obtain the result, while 5–10 min is needed for the Schirmer test per eye. In addition, the thread size is smaller than that of Schimer’s strip. Therefore, it is suitable for experimental animals, including mice [152]. The strip/thread was inserted over the inferior lid margin toward the lateral canthus for five min. The eyes are allowed to blink normally. The wetting length was measured in millimeters after an equal selection time for all animals.

#### 4.2.2. Tear Breakup Time

The TBUT test is a specific test to evaluate the quality of the tear film. In DED, tear film becomes unstable due to tear deficiency that may cause either a lack of tear production or increase in the evaporation of tears due to environmental factors, lack of MBG secretion, or mucin. Therefore, tear film breaks quicker in DED than in normal eyes. Usually, TBUT is ≤10 s for DED in mice [144], rats [141], and rabbits [145], and it may vary but is always lower than that in normal eyes. TBUT can be measured in two ways: by a slit lamp with a cobalt blue filter and by tear scope-plus (2413-P-2003, Keeler, UK) or Corneal Topographer (Carl Zeiss Meditec Ag, Oberkochen, Germany) [153]. For the former, one drop of 1% fluorescein eye drops was instilled into the lower conjunctival fornix. Then, the eyelids were blinked manually for one time, and then, the lids were held open. A slit lamp with a cobalt blue filter was used to evaluate the time required for the appearance of the first dark spot. This time is called the TBUT. On the other hand, noninvasive TBUT is measured by hand and held tear scope-plus. The eyelids were manually blinked to distribute the tear film; then, the eye was held open, and the time taken for distortion of the reflected image of the tear scope grid was recorded and considered as TBUT. In both methods, to obtain accurate results, the TBUT test was performed twice to obtain the average result.

### 4.3. Surface Structural Damage

Corneal fluorescein staining (CFS), Rose Bengal staining (RBS), and Lisamine green staining (LGS) are similar types of experiments used to evaluate the lesions of the ocular surface epithelium in DED (Table 8). Only damaged surface epithelial cells are stained by CFS and RBS, which is the principal mechanism of these methods. Therefore, as many cells are damaged, staining and darkness of staining are observed. Therefore, these techniques provide useful information about the severity of DED and the efficacy of therapeutic agents. Either CFS [66] or RBS [88,98] or LGS [112,146] can be selected for a particular experiment to avoid corneal tissue stress. However, two tests ((CFS and RBS) [67,79,83], (CFS and LGS) [133]) were also performed in one experiment. Three tests (CFS, RBS and LGS) were also performed in one experiment [114]. Even though two or three tests were performed on the same day, there was no discussion of the interval between the two experiments, and there was no information on washing after performing one test. Practically, this is time consuming, injurious to epithelial cells, and difficult to perform, as both tests are performed in the anesthetic state. Usually, 1% fluorescein sodium or 1% Rose Bengal stain or 1% Lisamine green stain is instilled (2–25 μL) into the conjunctival sac. Then, the eyelids were blinked manually for one time, and then, the lids were held open. After 3–10 min, cobalt blue light or red fluorescence staining or green staining, respectively, of the cornea was evaluated using a slit-lamp and scored.

There are various grading systems available for the evaluation of severity by staining, such as the van Bijsterveld scale (0–3, scoring from the whole cornea), National Eye Institute (NEI) scale (0–3, scoring from selected five zones), the Sjogren’s International Collaborative Clinical Alliance ocular staining score/ICCA OSS Group Scale (0–3, scoring from the whole cornea), Baylor scale (0–4, scoring from selected five zones), and Oxford scale (0–5, scoring from whole corneas) [154]. Among them, Oxford scale scoring and NEI scales are the most popular in experimental studies. However, the Oxford scale scoring system could be convenient for statistical analysis and comparing and finding statistical significance among experimental groups (Table 9).

### 4.4. Analysis of Molecular Changes and Histopathological Changes

DED is a chronic inflammatory disease. Therefore, evaluation of inflammatory cytokines in the ocular surface epithelial (epithelium of cornea or conjunctiva) cells, such as IL-1β, IL-8, TNF-α, and matrix metalloproteinases (MMPs), is the most common feature in experimental models [81,84,138,139,140]. Alongside the underlying stimulating factors of inflammation in the ocular surface, such as MAP kinase pathways (ERK, JNK, and P38) [140,148], NFkB has also been measured by many researchers. Since ocular surface stress and inflammation induce apoptosis, apoptosis-related factors BAX, BCl2, and their ratio were also measured [88]. As chronic DED fibrosis may induce apoptosis as well, fibrosis-related TGF-β1 [150] was also measured. Furthermore, angiogenesis in the ocular surface may be triggered by inflammation, so the angiogenesis-related factor VEGF-A was measured by Kwon J.W. et al. [139]. Endothelial cell damage with dislocation of ZO-1, and disruption of PAMR [63] (Table 8).

Hyperosmolarity stress and amplified inflammatory cascades in the ocular surface of DED significantly alter the thickness of the corneal epithelium to thinning. Therefore, the histologically most used parameter in experimental DED is the corneal epithelial thickness [79,81,84,88,147]. In DED, goblet cells were also reduced, which is responsible for mucin secretion. Conjunctival impression cytology (CIC) using hematoxylin and periodic acid-Schiff (PAS) reagent [79,148] or counting goblet cells in the conjunctiva using periodic acid-Schiff (PAS) staining [44,84]. Immunohistochemical slides were also stained with MUC5AC in the conjunctiva to count the goblet cells [79,83,138]. Furthermore, polymorphonuclear cells (PMNs) in the cornea and limbus by hematoxylin and eosin stains were evaluated to measure the infiltration of inflammatory cells [138].

## 5. Conclusions

**Figure 3:**
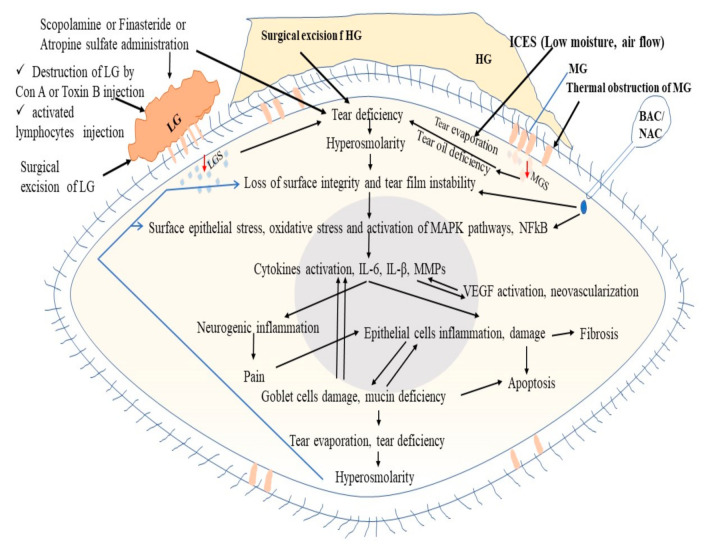
Mechanisms of various experimental methods for induction of dry eye disease. LG, lacrimal gland; LGS, lacrimal gland secretion; HG, Harderian gland; MBG, Meibomian gland; ICES, Intelligently controlled environmental system; BAC, Benzalkonium chloride; NAC, N-acetylcysteine; MAPK, Mitogen-activated protein kinase; NFkB, Nuclear factor kappa B; IL-6, Interleukin 6; IL-1β, Interleukin-1beta; MMPs, Matrix metalloproteinases; VEGF, Vascular endothelial growth factor.

Tear hyperosmolarity is a key factor responsible for DED. Tear hyperosmolarity induces loss of surface integrity, tear film instability, and surface epithelial stress, which triggers the inflammatory cascades via MAP kinase and NFkB signaling pathways. This inflammation is responsible for multifactorial clinicopathological outcomes, such as epithelial cell damage, apoptosis, pain, fibrosis, and neovascularization (Figure 3, Table 8). Tear hyperosmolarity may be induced by a lack of lacrimal secretion called tear or higher tear evaporation due to abnormality of MBG secretion, goblet cell secretion, or rough environmental factors (low moisture, higher airflow). In an experimental study, the administration of scopolamine chloride, finasteride, atropine sulfate, and injection of Con-A or BTX-B activated lymphocytes to the LG or excision of LG and results in LGS deficiency, thereby inducing dry eye. BAC or NAC induces tear film instability, and the loss of the epithelium and mucus layer or mucin-like substances results in hyperosmolarity, which in turn leads to dry eye. Thermal obstruction of the MBG results in oil deficiency, such as susceptance, which results in tear evaporation and DED (Figure 3). Furthermore, the addition of 90 mM sodium chloride to the culture medium induced a hyperosmolarity environment and DED-like symptoms in the in vitro DED model, while the addition of benzalkonium chloride (0.0001% (1 μg/mL) was also used to induce inflammatory cascades, as in the in vivo experiment.

The animal models described above share similar features of DED in humans, and each model is an essential tool for investigating morphologic changes and underlying etiopathogenic molecular mechanisms that could also be involved during the evolution of DED. To develop new therapeutic drugs, it is necessary to test many agents in the preclinical research environment, and the use of smaller mammal models, such as mice or rats, will reduce the expense of producing test screening. Some advanced efficacy studies or toxicological examinations that require invasive procedures and large tear samples and tissue samples may be facilitated using animals with large body sizes, such as rabbits or non-human primates. In addition, the selection of a specific animal model depends on the type of drug being screened, the aim of the scientific strategy, investigator’s choice, institutional financial and facility resources in the DED research, and pharmaceutical drug discovery and development program. Importantly, the combination of two or three induction methods (genetic, chemical, or surgical methods with ICES) shortens the DED induction time, which is important for experimental research in saving time and money. In addition, the repetition of similar types of experiments, such as corneal surface staining tests, Schirmer’s test, and PRT test can be avoided for experimental schedule traffic and to relieve stress to animals and tissue injury.

## Figures and Tables

**Table 1:** Thickness of tear film in different animal.

Animal	Thickness	References
Mice	7 μm	Tran et al. [32] 2003, Johnson et al. [2] 2004
Gerbils	15 μm	Prydal et al. [33] 1993
	14 μm (interferometry) 16 μm (confocal microscopy)	Prydal et al. [34] 1992
Rat	2–6 μm	Chen et al. [31] 1997, Johnson et al. [2] 1992
	11 μm (interferometry) 11 μm (confocal microscopy)	Prydal et al. [34] 1992
	7 μm (glass fiber) 13 μm (confocal microscopy)	Prydal et al. [33] 1993
Rabbit	4–7 μm	Mishima [35] 1965, Prydal et al. [34] 1992
	12 μm (interferometry) 10 μm (confocal microscopy)	Prydal et al. [34] 1992
	7 μm (glass fiber) 10 μm (confocal microscopy)	Prydal et al. [33] 1993

**Table 2:** The available cell lines and primary cells, culturing methods, induction, and end point of in vitro DED experiment.

Name of Cells Line	Culture	Induction Methods	Starting of Treatment	End of Experiment	References
Wong Kilbourne derivative of Chang conjunctival epithelial cell line (WKD; clone 1–5c-4)	Dulbecco’s minimum essential medium supplemented with 10% fetal bovine serum, 1% glutamine, 50 UI/mL penicillin, and 50 UI/mL streptomycin	Cells were grown for 24 h. Then, benzalkonium chloride was dissolved in phosphate-buffered saline (PBS). Different concentrations of BAC (10−2%, 10−3%, were analyzed.	–	15 min of treatment or 15 min of treatment followed by 24 h of cell recovery in complete medium	Brasnu et al. [47] 2008
	Dulbecco minimum essential medium supplemented with 10% fetal bovine serum (FBS), 1% glutamine (200 mM stock solution), and 1% penicillin (10,000 units/mL) and streptomycin (10,000 μg/mL)	Cells were grown for 24 h using hyperosmotic media 500 mOsM, achieved by adding 90 mM sodium chloride or media containing benzalkonium chloride at 10–4%, 3.10–4%, or 5.10–4%.	–	Cell was analyzed at 24 h and 48 h	Clouzeau et al. [48] 2012
	Dulbecco’s minimum essential medium (DMEM) supplemented with 10% fetal bovine serum, 1% glutamine, 0.1% ampicillin, and 2% kanamycin	A 15 min BAC 0.001% treatment and after 15 min culture media was removed, and normal culture medium was added and allowed for 24 h	Candidate drug was mixed 1 h before BAC treatment	After 24 h	Debbasch et al. [49] 2001, Debbasch et al. [50] 2001
	Eagle’s minimal essential medium supplemented with 5% fetal calf serum, 2 mM L-glutamine, 50 mg/mL streptomycin, and 50 IU/mL penicillin	0.0001% (1 μg/mL). Cells were treated for 10 min. After this time, the BAC-containing medium was removed, cells were rinsed twice with culture medium, and normal cell culture conditions were restored.		Examined before treatment and 3, 24, 48, and 72 h later	De Saint Jean et al. [51] 1999
IOBA-NHC cells	DMEM/F12 supplemented with 1 μg/mL bovine pancreas insulin, 2 ng/mL mouse epidermal growth factor, 0.1 μg/mL cholera toxin, 5 μg/mL hydrocortisone, 10% fetal bovine serum (FBS), 50 UI/mL penicillin, and 50 UI/mL streptomycin	Cells were grown for 24 h. Benzalkonium chloride was dissolved in PBS. Different concentrations of BAC (10–2%, 10–3%, were analyzed.	–	Two incubation times were applied to the cells: 15 min of treatment and 15 min of treatment followed by 24 h of cell recovery in complete medium	Diebold et al. [52] 2003; Brasnu et al. [47] 2008
	Dulbecco’s Modified Eagle Medium (DMEM)/HAM’s F12 (1:1) supplemented with 10% fetal calf serum (FCS, Biochrom AG, Berlin, Germany) in a humidified incubator containing 5% CO2 at 37 °C	For stimulation, cells (1 × 106) were seeded in Petri dishes and cultured until confluence was reached. Cells were washed PBS and changed to serum-free medium for 3 h. Afterward, cells were either treated with recombinant proinflammatory cytokine interleukin (IL)-1β (10 ng/mL) or tumor necrosis factor (TNF) α (10 ng/mL) for 6 h, 12 h, 24 h, or 48 h	–	6 h, 12 h, 24 h, or 48 h each	Schiicht et al. [53] 2018
Human corneal epithelial cells (HCECs), a human transformed SV40 immortalized corneal epithelial cell line	Were cultured in Dulbecco’s modified Eagle’s medium/F12 with 10% fetal bovine serum and 10 ng/mL human epidermal growth factor and the medium replaced every other day.	Cells were grown for 24 h. Then, they were treated with a different osmolarity, ranging from 312 to 550 mOsm/L, which was achieved by adding 0, 70, 90, or 120 mM sodium chloride (NaCl) with or without candidate drugs.	Candidate drugs were added 2 h before adding NaCl.	Samples were after 24 h treatment	Li et al. [54] 2020
	Were cultured in Dulbecco’s modified Eagle medium (DMEM)/HAM’s F12 supplemented with 10% fetal bovine serum, 50 U/mL penicillin, 50 μg/mL streptomycin mixture, 1% insulin-transferrin–selenium mixture, and 10 ng/mL human epidermal growth factor	Hyperosmotic stress (500 mOsm) was achieved by adding 90 mM sodium chloride (NaCl, Sigma-Aldrich) to isosmotic medium (310 mOsm).	Candidate drugs were added 2 h before adding NaCl.	Supernatants of conditioned medium were collected at 24 h after stimulation	Ma et al. [55] 2021
Human conjunctival cell line HCC	Cells were cultured according to the manufacturer’s instruction in RPMI medium supplemented with 100 IU/mL penicillin, 100 mg/mL streptomycin, and 10% heat-inactivated FBS	Hyperosmotic media (528 mOsM)	Candidate drugs were added 2 h before adding NaCl.	24 h after treatment	Park et al. [56] 2019
**Primary Culture**					
Primary HCECs (human corneal epithelial cells) were cultured from donors within 72 h after death	Supplemented hormonal epidermal medium (SHEM) containing 5% FBS	The addition of 44, 69, and 94 mM of sodium chloride (NaCl) can achieve hyperosmolarity (400, 450, and 500 mOsM) from the isosmolar (312 mOsM) medium.	-	The HCECs co-incubated for 12 h, 24 h, or 48 h were used for immunostaining	Liu et al. [57] 2020
Primary culture of rabbit corneal epithelial cells (CECs) or Primary rabbit LG acinar cells (LGACs)	After isolation, cultured with DMEM/F12 (Dulbecco’s Modified Eagle Medium/Nutrient Mixture F-12) with 1% antibiotic–antimycotic solution	After 24 h culture, DED-like symptom was induced by the addition of IL-1β (10 ng/mL) with the medium.	Dexamethasone (10 μM) was used combined with IL-1β for treatment	Culture was maintained for 1 week and analyzed	Lu et al. [58] 2017

**Table 3:** Experimental protocol in chemically induced DED model.

Animal	Dose	Frequency	Days	Starting of Treatment	End of Experiment	References
Rabbits	0.01%, 0.05%, or 0.1% was applied	Twice daily to one eye	For 4 days			Chen et al. [44] 2011
Rabbits	0.1% BAC drops	Twice daily	For on days 5, 7, and 14		On days 5, 7, and 14	Xiong et al. [67] 2008
Rabbits	0.1% BAC	Twice daily	For 14 days	After 14 weeks of BAC treatment	3 days after treatment	Lu et al. [79] 2017
Rabbits	0.1% benzalkonium chloride (BAC) drops	Twice daily topical administration	For 3 weeks			Ji et al. [80] 2017
Rabbits	0.1% BAC (20 μL)	Thrice daily (10:00 a.m., 2:00 p.m., and 6:00 p.m.)	For 4 weeks	After 4 weeks of BAC treatment twice daily (10:00 a.m. and 6:00 p.m.) for 3 weeks	After 3 weeks of treatment	Tseng et al. [81] 2016
Rabbits	0.1% (wt/v) BAC (20 µL)	Thrice daily	For 4 weeks	After 4 weeks of BAC treatment	After 3 weeks of treatment	Chen et al. [82] 2017
Rabbits	0.1% BAC drops	Twice daily	For 2, 3, 4, or 5 weeks			Li et al. [66] 2012
Mouse	5 μL of 0.2% BAC	Twice daily (9:00 a.m., 9:00 p.m.)	For 7 days		On day 7	Lin et al. [83] 2011
Mice	5 μL of 0.1% BAC	Twice daily (9:00 a.m., 9:00 p.m.)	For 10 days	5 μL, three times per day (9:00 a.m., 3:00 p.m., 9:00 p.m.)	On day 6 of treatment	Xiao et al. [84] 2012
Mice	5 μL of 0.2% BAC	Twice daily (10:00 a.m. and 10:00 p.m.)	For 14 days	After 14 days BAC instillation	On the 14th days	Kim et al. [68] 2016
Mice	5 μL of 0.2% BAC	Twice daily (9:00 a.m., 9:00 p.m.)	For 14 days	After 14 days BAC treatment 5 μL, three times per day (8:00 a.m., 3:00 p.m., 10:00 p.m.)	On 6 days after treatment	Xiao et al. [85] 2012
Wister Rat	0.2% BAC	Twice a day	For 7 days	After 7 days of BAC treatment once daily for 1 week	After 7 days of treatment	Beyazyildiz et al. [86] 2014
SD rat	5 μL 0.2% BAC	Twice daily (at 7:00 a.m. and 7:00 p.m.)	For 7 days	–	On 7th day	Marques et al. [87] 2014
SD rat	0.2% BAC	Daily, at 9:30:00 a.m. and 5:30:00 p.m.	For 10 days	Immediately after BAC treatment	After 10 days	Na et al. [88] 2017
**Other Chemicals**						
**Atropine Sulfate**						
**Animal**	**Dose**	**Frequency**	**Days**	**Starting of Treatment**	**End of Experiment**	**References**
New Zealand adult female rabbits	One drop of 1% atropine	Three times daily 6:00 a.m., 2:00 p.m., and 10:00 p.m.	For 7 days	Oral administration of oils occurred at 8:00 a.m. from the first day with atropine sulfate to the 12 weeks	12 weeks	Silva et al. [74] 2017
White albino Rabbits	1% atropine sulfate	Three times daily	For 1 week	After 7 days atropine sulfate treatment for 7 days	On 7th day	Shafaa et al. [73] 2017
Rabbits	1% atropine sulfate	Three times daily	For 17 days	three times daily for seven days starting on day 10	On 17 days	El-Shazly et al. [89] 2008
Rabbits	50 μL of 1.0% atropine sulfate	Three times daily at 8:00 a.m., 1:00 p.m., and 6:00 p.m.	For 5 days.	–	–	Burgalassi et al. [72] 1999
New Zealand white rabbits	1 mg/kg atropine sulfate (1.0%).	Injected intramuscularly every day	For 3 days	–	After 3 days	Altinors et al. [90] 2007
**Finasteride**						
Wistar rats	Finasteride (1.16 mg/kg/d) was orally administered to all the rats	Once a daily	For 4 weeks	Same time once a day	4 weeks	Zhang et al. [71] 2016
Wistar rats	Finasteride (1.16 mg/kg/day) was orally administered to the rats	Once a day	For 4 weeks	–	–	Li et al. [91] 2018
Wistar female and male rats	Finasteride (1.16 mg/kg) was orally administered to all the female and male rats	Once a day	For 10 days	–	At the end of 10 days	Singh et al. [70] 2014
**N-Acetylcysteine (NAC)**						
SD rats	20 μL of 10% (wt/vol) NAC by topically	Four times (10:00 a.m., 12:00 p.m., 2:00 p.m., 4:00 p.m.) a day into the right eye of each rat	For 5 days			Li et al. [92] 2018
rabbit	a 10% (wt/vol) NAC solutions	instilled 6 times at 2 h intervals for 1 day (9:00 a.m. to 7:00 p.m.) into the eyes	For 1 day	Next day after NAC installation	2, 4, or 6 times a day for 3, 7, or 14 days	Urashima et al. [78] 2004
**Scopolamine**						
C57BL/6 mice	Subcutaneous injections of 0.1 mL of 5 mg/mL scopolamine hydrobromide	Three times daily	For 5 days		After 5 days	Xiao et al. [93] 2015
Female Lewis rats	Scopolamine was continuously and systemically delivered to the animals via an osmotic pump filled with scopolamine and implanted subcutaneously. In the first experiment, three doses of Scopolamine was delivered for 28 days, 12.5 mg/day.				At 28 days	Viau et al. [94] 2008
**Trichloroacetic Acid**						
New Zealand white rabbits	A cotton swab soaked with freshly prepared 50% trichloroacetic acid was applied to the conjunctivas of the left eyes 2–3 mm lateral to the corneal limbus	For 5 s (when blanching of the conjunctiva was observed). The conjunctival sacs were immediately washed with 100 mL 0.9% sterile saline				Li et al. [45] 2013

**Table 4:** Surgical methods of DED induction and experiment schedule.

Animal	Name of Parts	Starting of Treatment	End of Experiment	References
SD rats	The left exorbital lacrimal gland was surgically excised	At three days after surgery, orally administered for 7 days	After 7 days of treatment	Kang et al. [106]
C57BL/6 mice	Surgical excision of the left exorbital lacrimal gland	At three days after surgery 20 µL twice daily for 5 days	5 days after treatment	Kim et al. [107] 2016
Male Wistar rats	Surgical excision of the left exorbital lacrimal gland	At three days after surgery, 20 µL twice daily for 4 days	7 days after operation	Park et al. [108] 2018
C57BL/6 mice	Dry eye based on severe aqueous fluid deficiency, by excising both the exorbital and intraorbital lacrimal glands of mice.	–	8 weeks	Shinomiya et al. [14] 2018
Squirrel Monkey	Unilateral removal of main lacrimal gland.		20 weeks	Maitchouk et al. [95] 2000
BALB/c mice	After anesthesia, 10 or 20 μL concanavalin A (ConA) that was diluted in phosphate-buffered solution (PBS) at concentrations of 10 mg/mL was injected into the intraorbital gland through a transconjunctival approach using a Hamilton syringe with a 33-gauge needle under an operating microscope	Immediately after ConA injection, hMSCs (1 × 103 or 1 × 105 cells/20 μL BSS), mMSCs (1 × 105 cells/20 μL BSS), or the same volume of BSS were injected into the periorbital space using a 30-gauge needle syringe	After 7 days	Lee et al. [101] 2015
BALB/c mice	20 µL ConA that was diluted in PBS at the concentration of 10 mg/mL was injected into both intraorbital and extraorbital lacrimal glands of BALB/c mice using a Hamilton syringe with a 33-gauge needle	Recombinant human (rh) TSG-6 (1 µg/10 µL was topically instilled four times a day (QID) to the ocular surface of the mice for 7 days	After 7 days of treatment	Lee et al. [102] 2015
Balb/c mice	Dry eye disease was induced using 10 mg/mL of ConA (20 µL) in PBS, which was injected into the lacrimal glands with a 28.5 gauge needle using a dissecting microscope	Individually combined together and injected with ConA	After 7 days of treatment	Ratay et al. [100] 2017
NZW rabbit	After anesthesia, rabbits were injected with 500 μg of Con A in 50 μL of saline in the lacrimal glands bilaterally using a 26-gauge needle	24 h after Con A injection, ophthalmic solution four times a day for 6 days	48 h after last Con A injection	Seo et al. [103] 2010
NZW rabbit	A single 30 μL volume 300 μg Con A was injected into the lacrimal gland using a 30-gauge needle and a Hamilton syringe	Injected combined with ConA	7 days after treatment	Zheng et al. [104] 2015
New Zealand albino rabbits	Lacrimal glands and Meibomian glands. The Harderian		After 10 weeks	Polans et al. [96] 2017
Japanese albino rabbits	The lacrimal and harderian glands and nictitating membrane were removed surgically		4 months after surgery	Chen et al. [44] 2011
New Zealand white rabbits weighing	The lacrimal gland, Harderian gland, and nictitating membrane of the left eyes were surgically removed		On day 56	Li et al. [45] 2013
New Zealand white rabbits	Nictitating membrane (NM), Harderian gland (HG), and main LG		4 months after excision	Bhattacharya et al. [43] 2015
New Zealand white rabbits	Resection of main LG, HG, and NM			Ning et al. [42] 2016
CBA/J mice	Injection of 0.05 mL of 20-mU BTX-B solutions into the left lacrimal gland	3 days after BTX injection	4 weeks after BTX injection	Lekhanont et al. [105] 2007

**Table 5:** Environmental factors-induced DED model and experiment schedule.

Animal	Humidity	Airflow	Temperature	Time of Induction	Starting of Treatment	End of Experiment	References
C57BL/6 mice	13.1% ± 3.5%	2.2 ± 0.2 m/s	22 ± 2 °C	21 days	Beginning of housing in the ICES or on day 22 after DED confirmation. 10 µL topically, four times daily until 35 days.	35 days	Chen et al. [115] 2013
BALB/c mice	13.1% ± 3.5%	2.2 ± 0.2 m/s	22 ± 2 °C	21 days	After 21 days housed in the ICES, the mice were administered 10 μ of eye drops, four times daily (every 6 h) for 14 days during which the mice remained housed in the ICES.	35 days	Chen et al. [116] 2009
C57BL/6 mice	13.1% ± 3.5%	2.2 ± 0.2 m/s	22 ± 2 °C	21 days	10 μL/eye bilaterally four times a day for 3 weeks.	After 3 weeks	Li et al. [117] 2012
C57BL/6 mice	15.3% ± 3.0%	2.1 ± 0.2 m/s	21 °C ± 2 °C	14 days	Beginning of housing in the ICES or on day 14 after DED confirmation. 10 µL topically, 4 times daily until 28 days	28 days	She et al. [109] 2015
BALB/c mice	15.3% ± 3%	2.1 ± 0.2 m/s	21 ± 3 °C	42 days	–	42 days	Chen et al. [110] 2008
C57BL/6 mice	13.1 ± 3.5%, airflow and temperature	2.2 ± 0.2 m/s	22 ± 2 °C	An alternating 12 h light–dark cycle (8:00 a.m. to 8:00 p.m.) was employed for 1, 2, 4, and 6 weeks		1, 2, 4, and 6 weeks	Xiao et al. [93] 2015
Mice	Topical application of:00 PM2.5. 5.0 mg/mL, 4 times daily					4, 7, 10, and 14 days	Tan et al. [113] 2018
Mice	Topical application of PM10 5.0 mg/mL to right eyes, 4 times daily.					At 14 days	Li et al. [114] 2017
Rats	Rats were exposed to approximately 500 μg/m^3^ UPM in the exposure chamber for 5 h per day over 5 days.					5 days after exposure of UPM	Song et al. [112] 2020

**Table 6:** Some selected genetic mouse models and time point DED-related symptoms.

Animal	Time of Induction	Detected Symptoms Related to DED	References
TGF-β1 knockout mouse	2 and 4 weeks of age	Significant inflammatory cell infiltrates in the lacrimal gland between the ages of 2 and 4 weeks	McCartney Francis et al. [123] 1997
NOD. Aire KO mice	6 weeks of age	Severe corneal pathology observed	Vijmasi et al. [124] 2013
NRTN−/− mice	6 weeks of age	Tear volume and mucin production are decreased, and ocular surface inflammation are increased	Cha et al. [121] 2002
C57BL/6.NOD-Aec1R1Aec2,	19–22 weeks of age	Male mice displayed a high level of dacryoadenitis	Nguyen et al. [125] 2006
NOD.B10.H2b	12 weeks of age	Severe corneal pathology observed	Lee et al. [75] 2017, Kim et al. [76] 2015, Lee et al. [102] 2015
TSP-1−/− mice	6–12 weeks of age	6–8 weeks, changes of LG epithelial cells and their functional loss are observed At 12 weeks, the loss of corneal surface integrity and corneal nerve morphology, conjunctival infiltrates, and loss of conjunctival goblet cells	Masli et al. [120] 2020
IL-2Rα (CD25) knockout mice	8 weeks of age	CD4+ cells are detected in the conjunctiva beginning at 8 weeks of age and disrupted only from 12 weeks onwards.	Masli et al. [120] 2020
NFS/sld mice	8 weeks of age	At 8 weeks, inflammatory lesions of lacrimal glands and Harderian glands, loss of ocular surface integrity, and conjunctival goblet cells observed.	Arakaki et al. [126] 2014
IQI/Jic mouse	9 months of age	After 21 days housed in the ICES, the mice were administered 10 μ of eye drops, four times daily (every 6 h) for 14 days, during which the mice remained housed in the ICES.	Chen et al. [127] 2009

**Table 7:** Application of combined methods and experimental schedule in DED model.

Animal	Name of Chemical/Surgery	Environmental Condition/Other	Starting of Treatment	End of Experiment	References
C57BL/6 mice	Scopolamine hydrobromide (0.5 mg/0.2 mL) was injected subcutaneously in the dorsal skin of mice three times per day.	Exposed to a relative humidity < 25%, temperature of 20–22 °C, and airflow of 15 L/min, 24 h per day	-	On day 7 or 9	Lee et al. [75] 2017, Lee et al. [128] 2012
C57BL/6 mice	In brief, 0.5 mg/0.2 mL scopolamine hydrobromide was injected subcutaneously in the dorsal skin of mice three times daily.	The mice placed in the CEC were continuously exposed to a relative humidity < 30%, a constant temperature of 21–23 °C, and airflow of 15 L/min, 24 h a day	From day 3 to day 9, 2 µL of the topical agent was applied to both eyes of each mouse, twice a day (9:00 a.m. and 5:00 p.m.).	On day 9	Lee et al. [129] 2011
C57BL/6 mice	Topical application of atropine sulfate, 1%, twice daily for the first 48 h. In addition, the mice also received subcutaneous 0.1-mL injections of 5 mg/mL scopolamine hydrobromide three times a day (9:00 a.m., 1:00 p.m., and 5:00 p.m.) on their dorsal surface for the duration of the experiment.	Regulation of relative humidity <30%, <30%, a constant temperature of 21–23 °C, and airflow of 15 L/min, 24 h a day	48 h after the induction of dry eye, 3 µL of the topical formulatio nwas applied to both the eyes of the unanesthetized mice, twice a day (9:00 a.m. and 5:00 p.m.) from days 3 to 9.	9 days of treatment	Goyal et al. [130] 2009
C57BL/6 mice	Scopolamine was injected into the dorsal skin of mice (0.5 mg/0.2 mL at 9:00 a.m., 12:00 p.m., and 3:00 p.m.; 0.75 mg/0.3 mL at 6:00 p.m.).	Mice were placed in the controlled environmental chamber (relative humidity <30%, airflow 15 L/min, temperature 21–23 °C)	1 µL of eye drop was applied topically to the eye of an unanesthetized mouse once daily from 48 h to day 4 (total three doses) or day 9 (total eight doses).	On day 5 or 10 after treatment	Rashid et al. [131] 2008
C57BL/6 mice, and BALB/c	DS was induced by subcutaneous injection of scopolamine hydrobromide (0.5 mg/0.2 mL; four times a day (at 08:00 a.m., 12:00 p.m., 2:00 p.m., and 5:00 p.m.), alternating between the left and right flanks of 4–6-week-old mice	Mice were placed in a cage with a perforated plastic screen on one side to allow airflow from a fan (Cafrano) placed 6 inches in front of it for 16 h/day for 5 or 12 consecutive days; chamber (relative humidity 30–35%, airflow 15 L/min, temperature 80 °F)	–	On day 12	Niederkom et al. [132] 2006
129SvEv/CD-1 white mice	Subcutaneous injection of scopolamine (1 mg in 0.2 mL) three times daily in the flanks of 4–6-week-old 129SvEv/CD-1 white mice. Dry eye was induced in mice with a modification of a previously described technique.	Mice were exposed to a continuous air draft from a fan placed 15.24 cm in front of the cage in an environmentally controlled room (50% humidity, 18 °C) for 10 h a day for 12 consecutive days	–	On day 12	Yeh et al. [134] 2003
C57BL/6 and IFN- γ knockout mice	Subcutaneous injection of scopolamine hydrobromide (0.5 mg per 0.2 mL four times a day (8:00 a.m. 12:00 p.m., 2:00 p.m., and 5:00 p.m.), alternating flanks of mice	Humidity was maintained at 30–35% 16 h per day. DS was induced for either 5 or 10 consecutive days			De Paiva et al. [135] 2009
Male NOD.B10.H2b mice	0.5 mg/0.2 mL hypodermic injection of scopolamine hydrobromide into both hindquarters (one after the other) four times (9:00 a.m., 12:00 p.m., 3:00 p.m., and 6:00 p.m.) per day for 10 days	Desiccation stress was created by low ambient humidity (30–40%) using an air draft from a fan for 18 h per day	After the desiccation stress for 10 days, the scopolamine hydrobromide injections were discontinued, and the mice were placed in an environment of normal humidity and temperature. The eye drops, PBS, and 1 mg/mL or 5 mg/mL silk fibroin were administered five times (9:00 a.m., 11:00 a.m., 1:00 p.m., 3:00 p.m., and 5:00 p.m.) per day for 10 days	After 10 days of treatment	Kim et al. [122] 2017
New Zealand rabbits	Surgical dissection of nictitating membrane	After 1 week of surgery, animal were housed in controlled environment humidity, 22% ± 4%, air flow 3 to 4 m/s, and temperature 23 to 25 °C	–	After 3, 7, and 14 days	Chen et al. [133] 2021

**Table 8:** Evaluation methods and parameters in experimental DED.

Types of Tests	Items	Animal	Methods	References
Clinical sings		Rabbit	Conjunctivitis (0–3), ocular discharge (0–3), corneal opacity (0–3)	Baudouin et al. [l] 2013, Silva et al. [74] 2017
	Modified severity score from Eaton J.S. et al. [137]; Silva D.A. et al. [74]		The scored varies from 0 to 16 and was defined as the sum of the individual scores graded from conjunctival hyperemia (0–3), conjunctival swelling (chemosis) (0–4), conjunctival discharge (0–3), corneal opacity (area) (0–4), and corneal vascularization (0–2).	Silva et al. [74] 2017, Eaton et al. [137]
Inflammatory index/scoring and markers	Macroscopic (visually) inflammatory scoring/index	Mice	Total score of 0–9; ciliary hyperemia (0–3); central corneal edema (0–3); peripheral corneal edema (0–3)	Lin et al. [83] 2011, Xiao et al. [84] 2013, De Paiva et al. [135] 2009, Laria et al. [136] 1997
	Microscopically evaluated by infiltration of inflammatory cells	Rats	Evaluated by counting PMN cells in the cornea and limbus by HE stains	Han et al. [138] 2017
	Inflammatory cytokines	Mice, Rat	TNF-α, IL-6, TNF- α, IL-1 a, IL-1β, and MMP-9 in corneas, CD11b+ (IHC)	Xiao et al. [84] 2012, Han et al. [138] 2017, Kwon et al. [139] 2016, De Paiva et al. [140] 2006
		Rabbit	TNF-α, IL-1 β, IL-6, IL-8 in corneas	Tseng et al. [81] 2016
		Rabbit	IL-1β, TNF-a, and MMP-9 in conjunctiva epithelium	Bhattacharya et al. [43] 2015
Test for detection of tear abnormality	Tear film osmolarity	Rats	Using Tearlab Osmolarity System^®^ osmometer. Osmolarity increase in DED.	Marques et al. [87] 2014
	Schirmer’s I Test	Rabbit	Tear volume (mm)	Luo et al. [79] 2017, Tseng et al. [81] 2016
		Rats		Liu et al. [141] 2019
	Phenol red thread tests	Rabbit		Lin et al. [142] 2018
		Rat		Han et al. [138] 2017
		Mice		Dietrich et al. [143] 2019
	Tear breakup time (BUT) (Specific for tear film instability)	Mice, rats, rabbit	Usually, TBUT is ≤10 s for in DED	Liu et al. [141] 2019, Shinzawa et al. [144] 2019, Wei et al. [145] 2013
Surface structural damage	Corneal Fluorescein Staining Test	Rabbit		Luo et al. [79] 2017, Tseng et al. [81] 2016
		Rat		Liu et al. [141] 2019
		Mice	Oxford grading	Dietrich et al. [143] 2019
	Rose Bengal Staining Test	Rabbit		Luo et al. [79] 2017, Na et al. [88] 2017
		Rat		Beyazyildiz et al. [86] 2014
		Mice		Lin et al. [83] 2011
	Lisamine green staining	Rabbit		Chen et al. [133] 2021
		Rat		Song et al. [112] 2020
		Mice		Chen et al. [146] 2017
Changes of epithelial layers of cornea or conjunctiva	Corneal epithelial thickness	Rabbit		Luo et al. [79] 2017, Tseng et al. [81] 2016, Luo et al. [79] 2017, Tseng et al. [81] 2016
		Mice		Xiao et al. [84] 2013, Na et al. [88], Diego et al. [147] 2016
		Rat		Marques et al. [87] 2014
	Goblet cells count	Mice, Rabbit, Rats	Counting the goblet cells number by staining MUC5AC in the conjunctiva	Luo et al. [79] 2017, Lin et al. [83] 2011, Han et al. [138] 2017
		Rabbit	Conjunctival impression cytology (CIC) by hematoxylin and periodic acid-Schiff (PAS) reagent	Luo et al. [79] 2017, Jiang et al. [148] 2017
		Rabbit, mice	Counting goblet cells in conjunctiva by periodic acid-Schiff (PAS) staining	Chen et al. [44] 2011, Xiao et al. [84] 2013
Molecular analysis	MAP kinase pathways	Rabbit	*p*-ERK1/2 protein expression	Jiang et al. [148] 2017
		Mice	phospho-JNK/total JNK, phospho-ERK/total ERK, phospho-p38 (*p*-p38)/total p38 (*p*-38) in the corneal epithelia	De Paiva et al. [140] 2006
	NFkB	Mice, HCE cells	Western blot analysis of NFkB	Tan et al. [113] 2018, Zhao et al. [149] 2019
	Apoptosis	Rabbit, mice, rat	TUNEL assay (apoptotic cells count)	Tseng et al. [81] 2016, Luo et al. [79] 2017, Xiao et al. [84] 2013, Han et al. [138] 2017
		Mice	Bax, BCL2, Bax/BCL2	Na et al. [88] 2017
	Vascular dysfunction	Mice	VEGF-A in corneas; cell vascular endothelial cells on corneal flat mounts	Kwon et al. [139] 2016
		Rabbit	endothelial cell damage with dislocation of ZO-1, and disruption of PAMR	Liang et al. [63] 2008
	Fibrosis	Rabbit	TGF-β1	Yao et al. [150] 2010

**Table 9:** Ocular surface scoring methods by the Oxford scoring system (Bron et al. [155]).

Image Panel	Verbal Description	Dot Count	Grade and Criteria
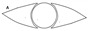	Absent	0 or 1	0 (=Panel A or < Panel B)
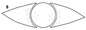	Minimal	10	1 (≤Panel B or < Panel C)
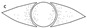	Mild	32	2 (≤Panel C or < Panel D)
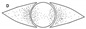	Moderate	100	3 (≤Panel D or < Panel E)
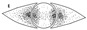	Marked	316	4 (≤Panel E or < Panel F)
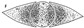	Severe	>316	5 (< Panel E)

Scoring system: If counted stained dot spot is 0-1, score is 0; if dot spots in-between 1-10, score 1; if dot spots in-between 11-32, score 2; if dot spots in-between 33-100, score 3; if dot spots in-between 101-316, score 4; if dot spots are more than 316, score is 5.

## Data Availability

Not applicable.
